# Peritoneal lymphomatosis confounded by prior history of colon cancer: a case report

**DOI:** 10.1186/1471-2407-11-276

**Published:** 2011-06-27

**Authors:** Yun Gi Kim, Ji Yeon Baek, Sun Young Kim, Dong Hyeon Lee, Weon Seo Park, Youngmee Kwon, Min Ju Kim, Jeehoon Kang, Joo Myung Lee

**Affiliations:** 1 Department of Internal Medicine, Seoul National University College of Medicine, Seoul, Republic of Korea; 2 Center for Colorectal cancer, Research Institute and Hospital, National Cancer Center, Goyang, Gyeonggi, Republic of Korea; 3 Department of Pathology, Research Institute and Hospital, National Cancer Center, Goyang, Gyeonggi, Republic of Korea

## Abstract

**Background:**

It is well known that carcinomas of the gastrointestinal tract are frequently associated with peritoneal carcinomatosis. In contrast to that entity extensive involvement of the peritoneal cavity with malignant lymphoma is rare.

**Case presentation:**

This is the first case reporting coexistence of peritoneal lymphomatosis and a previous history of colon cancer, which is a highly challenging clinical situation.

**Conclusions:**

If not aware of this unusual condition medical history, radiologic finding and laboratory data alone can lead to wrong diagnosis as in this case.

## Background

Peritoneal lymphomatosis is an extremely rare presentation of lymphoma. It receives much less attention than peritoneal carcinomatosis in clinical practice due in part to its relative low frequency [1]. It is difficult to differentiate between lymphomatosis and carcinomatosis, as well as peritoneal tuberculosis or other pathologic entities within the peritoneal cavity based on clinical and radiologic features [2]. Due to different potential curability and chemotherapeutic intervention, peritoneal lymphomatosis should be differentiated from peritoneal involvement of metastatic carcinoma or other diseases [3,4]. This is a case report of a diffuse large B-cell lymphoma initially presenting with peritoneal lymphomatosis and ascites in a patient with a history of colon cancer.

## Case presentation

An 81-year-old-man was admitted to the emergency department on August 2, 2010 for an acute onset of abdominal pain. Loss of appetite and general weakness had started a few weeks earlier, getting worse recently. No fever was noted at home. Physical examination showed multiple purpura on the trunk and both lower extremities with gum bleeding. There was no enlargement of lymph nodes and organomegaly by palpation. In his medical records, he underwent right hemicolectomy and partial duodenopancreatectomy with splenectomy for stage IIIC (pT4bN2aM0, restaging by the American Joint Committee on Cancer staging system, 7 th edition) ascending colon cancer in July 2006. He received adjuvant chemotherapy and was regularly followed as recommended by the National Comprehensive Cancer Network practice guidelines and was free of disease until March 2010.

**Figure 1:**
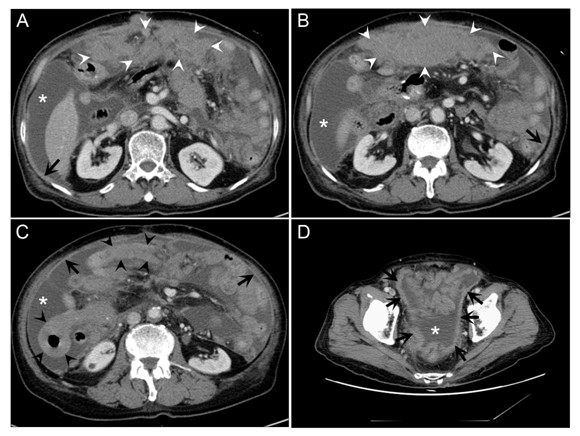
**81-year-old man with a peritoneal lymphomatosis by diffuse large B-cell lymphoma**. CT scan images (A-D) show diffuse peritoneal thickening (black arrows), diffuse nodular infiltration of omentum (white arrowheads), bowel wall thickening (black arrowhead) and ascites (asterisk).

Under the impression of colon cancer recurrence, an abdominal CT scan was performed. The CT scan revealed massive ascites and thickening of peritoneum and bowel walls, which was compatible with peritoneal carcinomatosis. (Figure 1) His complete blood cell counts showed hemoglobin level of 12.5 g/dL, total leukocyte count of 15 × 10^9^/L with 70% neutrophils, 15% lymphocytes, 9% monocytes, 4% eosinophils; and platelet count of 11 × 10^9^/L. Blood biochemical test results were unremarkable. While evaluating the evidence of recurrence, ascites tapping was performed for both diagnostic and palliative purposes, with the following results: ascites white blood cell count of 52,210/mm^3^, protein level of 2,600 mg/dL, albumin level of 2.5 g/dL (serum albumin, 3.5 g/dL) and serum-ascites albumin gradient of 1.0 g/dL. The patient was diagnosed with peritoneal carcinomatosis and was transferred to a local hospital for palliative care.

**Figure 2:**
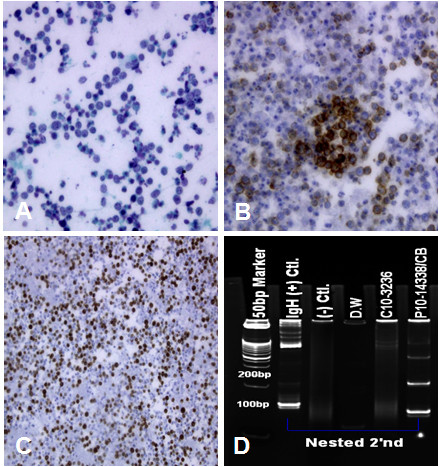
**Cytologic features of ascites**. A) Malignant lymphoid cells with large hyperchromatic nuclei and high nuclear/cytoplasmic ratio are evident. (x400). B) Tumor cells express B cell marker, CD20. C) Tumor cells demonstrate high Ki67 index. D) Immunoglobulin heavy chain rearrangement study demonstrate clonal rearrangement. IgH(+)Ctl; positive control, (-)Ctl; negative control; D.W; distilled water, C10-3256; case with negative control, p10-14338/CB; present case

After 7 days, cytology of ascites showed atypical lymphoid cells. Additional lymphocyte surface markers by immunohistochemical and molecular studies revealed positive results for CD79a, CD20 and Ki-67 (80%), but negative for CD3, suggesting a malignant lymphoma of B-cell lineage. (Figure 2) LDH levels of his serum and ascites were elevated up to 1,866 U/L and 2,200 U/L, respectively (normal value, 101-202 U/L). Based on these findings, he was diagnosed with diffuse large B-cell lymphoma presenting with peritoneal lymphomatosis and ascites.

After informing the patient and his family of the final diagnosis and the possibility that diffuse large B cell lymphoma may better respond to chemotherapy, the patient visited the ER once again. One day after his admission to ER, he had refractory metabolic acidosis due to tumor lysis syndrome, and he began continuous renal replacement therapy. Because of difficulties in patient positioning and the family's concern about possible bleeding complication, bone marrow biopsy could not be performed. The patient underwent dexamethasone treatment followed by R-CVP considering his poor performance status, but he died due to respiratory complications and multi-organ failure one month after he was diagnosed with peritoneal lymphomatosis. An autopsy could not be performed due to family's refusal. The refractory thrombocytopenia was thought to be the result of bone marrow involvement. If we had suspected the possibility of peritoneal lymphomatosis and closely communicated with pathologist, 3 to 4 days might have been saved and an appropriate therapeutic intervention could have begun before the patient's condition deteriorated.

## Conclusions

Peritoneal lymphomatosis is an unusual condition and is highly indistinguishable from peritoneal carcinomatosis. Differentiating peritoneal lymphomatosis from peritoneal carcinomatosis or tuberculous peritonitis by imaging modality is virtually impossible [5]. Lynch MA *et al. *reported that CT findings of 7 patients with peritoneal lymphomatosis closely mimicked diffuse carcinomatosis [9]. Devrim *et al. *also reported that CT findings of 16 patients with peritoneal lymphomatosis were highly indistinguishable from peritoneal carcinomatosis [10]. Recently, Lu SJ *et al. *reported positron emission tomography/computed tomography scanning finding of a single patient which showed diffusely increased metabolic activity in the peritoneum [11]. However, there is no systematic review of positron emission tomography/computed tomography scanning findings of peritoneal lymphomatosis until now. Regarding their effective medical treatment, an early differential diagnosis may encourage patients to receive treatment.

Clinically, it is always a great challenge to diagnose peritoneal lymphomatosis, and there are several case reports describing the diagnostic delay and confusion. Weng *et al. *[7] reported a case of peritoneal lymphomatosis, in which a correct diagnosis was delayed until the final pathology report due to the patient's high ascites ADA level mimicking peritoneal tuberculosis. A case report by Horger *et al. *[6] presented a case of a 32-year-old woman whose elevated CA-125 level and right ovarian enlargement led to an incorrect presumptive diagnosis of ovarian carcinoma with peritoneal carcinomatosis. This is a case in which a history of colon cancer led to an incorrect presumptive diagnosis and a delay in initiation of adequate treatment. There is no medical literature presenting peritoneal lymphomatosis with a history of colon cancer, a highly challenging clinical situation. Surprisingly, the current case showed elevated levels of serum CA-125 (196 U/L), which was similar to a finding reported by Horger *et al. *[6] Our patient also had elevated level of ADA (45 U/L), but with much lesser extent than that of Weng *et al. *[7] (695 U/L)

Previous reports showed extremely elevated levels of ascites LDH [6,7]. Weng *et al. *[7] reported his patient's ascites LDH as high as 2001 U/L, and Horger *et al. *[6] 1800 U/L. In this case, LDH levels of serum and ascites were 1866 U/L and 2200 U/L, respectively. This patient had no medical history or physical findings compatible with peritoneal lymphomatosis, and showed no discernable primary site. There was no clue to suspect peritoneal lymphomatosis except elevated ascites and serum LDH levels. It is well known that serum LDH level is elevated in many forms of lymphoma [8], but there is no clinical study which defined a relationship between peritoneal lymphomatosis and elevated ascites LDH level. This case and aforementioned case reports [6,7] suggest that ascites LDL level is well elevated in peritoneal lymphomatosis and is an important clue for differential diagnosis.

A confirmatory diagnosis should be made by cytologic examination of ascites or pathologic examination of peritoneal biopsy. As described above, it is unwise to make a differential diagnosis of peritoneal seeding and ascites only with medical history, conventional imaging and laboratory data. Early confirmative diagnosis through cytologic or pathologic examination can avoid a delay in treatment for peritoneal lymphomatosis. At the very least, it seems wise to simply check serum and ascites LDH levels before making the final diagnosis. One limitation in diagnosing lymphomas by effusions or ascites is the inability to analyze lymph node architecture. With the help of ancillary studies, such as flow cytometry and immunohistochemical staining or molecular workup such as gene rearrangement study, an accurate diagnosis can now be made in the majority of cases [7].

Diffuse large B-cell lymphoma presenting with peritoneal lymphomatosis is rare. This case suggests that peritoneal lymphomatosis warrants further investigation even in patients whose medical history strongly suggests peritoneal carcinomatosis. DLBCL can initially manifest as a form of peritoneal lymphomatosis with bone marrow involvement without solid tumor component as in this case.

## Abbreviations

CT: computed tomography; CEA: carcinoembryonic antigen; LDH: lactic dehydrogenase; R-CVP: rituximab-cyclophosphamide, vincristin and prednisolone; DLBCL: diffuse large B-cell lymphoma; ADA: adenosine deaminase

## Competing interests

The authors declare that they have no competing interests.

## Consent

Written informed consent was obtained from the patient's son for publication of this case report and any accompanying images. A copy of the written consent is available for review by the Editor-in-Chief of this journal.

## Authors' contributions

The original manuscript was written by YGK. All authors contributed to drafting and editing the manuscript. All authors read and approved the final manuscript.

## Pre-publication history

The pre-publication history for this paper can be accessed here:

http://www.biomedcentral.com/1471-2407/11/276/prepub
